# Training system for laparoscopy-assisted distal gastrectomy

**DOI:** 10.1007/s00595-016-1439-9

**Published:** 2016-11-09

**Authors:** Shinji Kuroda, Satoru Kikuchi, Naoto Hori, Shuichi Sakamoto, Tetsuya Kagawa, Megumi Watanabe, Tetsushi Kubota, Kazuya Kuwada, Michihiro Ishida, Hiroyuki Kishimoto, Futoshi Uno, Masahiko Nishizaki, Shunsuke Kagawa, Toshiyoshi Fujiwara

**Affiliations:** 1 Department of Gastroenterological Surgery, Okayama University Graduate School of Medicine, Dentistry and Pharmaceutical Sciences, 2-5-1 Shikata-cho, Kita-ku, Okayama, 700-8558 Japan; 2 Department of Surgery, Shobara Red Cross Hospital, Shobara, Japan; 3 Department of Surgery, Tsuyama Chuo Hospital, Tsuyama, Japan; 4 Department of Surgery, Hiroshima City Hiroshima Citizens Hospital, Hiroshima, Japan; 5 Department of Surgery, Okayama Saiseikai General Hospital, Okayama, Japan

**Keywords:** Laparoscopy-assisted distal gastrectomy, Gastric cancer, Training system, Learning curve

## Abstract

**Purpose:**

Laparoscopy-assisted distal gastrectomy (LADG) is likely to become a standard procedure for gastric cancer, which highlights the importance of establishing a training system in which even inexperienced surgeons can perform this procedure safely. This study assesses our training system for LADG based on short-term surgical outcomes.

**Methods:**

We evaluated retrospectively the short-term outcomes of 100 consecutive LADGs with curative D1/D1+ lymph node dissection. Our training system was assessed based on the learning curve of trainees, and factors related to achieving good-quality operations were analyzed statistically.

**Results:**

Overall, postoperative complications developed in 10 patients (10%), and included one case of anastomotic leakage (1%) and one case of pancreatic fistula (1%). The learning curve of the trainees plateaued after 10 operator cases in terms of operation time. The importance of the trainer’s position was also confirmed by the result that the operation time was significantly longer when trainees with ≤10 operator cases performed LADG with a trainer as scopist vs. a trainer as the first assistant. Univariate and multivariate analyses revealed that >10 operator cases were the most important factor for achieving good-quality operations.

**Conclusion:**

These results show that our current LADG procedure and training system are appropriate and effective.

## Introduction

Laparoscopy-assisted distal gastrectomy (LADG) has been developing steadily since first reported in 1994, because it is so minimally invasive and hence, characterized by less operative blood loss, less pain, earlier recovery of bowel activity, and a shorter hospital stay, than the conventional open procedure [1, 2]. The latest Japanese gastric cancer treatment guidelines describe LADG as an option in general clinical practice for clinical stage I cancer [3]. The safety of LADG for clinical stage I gastric cancer was recently confirmed in terms of the incidence of anastomotic leakage or pancreatic fistula formation, in a multicenter, phase II clinical trial conducted by the Gastric Cancer Surgical Study Group of the Japan Clinical Oncology Group (JCOG 0703) [4]. However, in this trial, all procedures were performed by surgeons with high credentials, because LADG is technically a more complicated and advanced procedure than open gastrectomy, and very challenging for inexperienced surgeons and hospitals.

The Endoscopic Surgical Skill Qualification System (ESSQS) was established by the Japan Society for Endoscopic Surgery (JSES) in 2001 with the aim of improving endoscopic surgical techniques and standardizing endoscopic surgery [5]. Endoscopic surgeons qualified by the ESSQS are required not only to endeavor to advance their own surgical skills, but also to train beginners and medical staff in endoscopic surgery. A successful training system for endoscopic surgery was reported through evaluation of the learning curve of operation time and blood loss. Standardization of the whole process of endoscopic surgery is emphasized as an important factor to achieve rapid progress [6–10].

We introduced LADG with curative lymph node dissection for the early gastric cancer to our hospital in 2007, since when we have tried to establish a standardized procedure involving not only the operator, but also a first assistant and scopist as a team for LADG after the arrival of an ESSQS-qualified surgeon in our hospital in October 2010. We conducted this study first to evaluate the safety and feasibility of LADG with curative lymph node dissection for early gastric cancer performed in our hospital. Then, our training system and standardized procedure for LADG were assessed through that evaluation, and factors related to achieving good-quality operations and smooth technical progress were analyzed further.

## Materials and methods

### Patients

We reviewed the medical records of 100 consecutive patients with gastric cancer, who underwent LADG with curative D1 or D1+ lymph node dissection according to the latest Japanese gastric cancer treatment guidelines, between October, 2010 and October, 2014, in our hospital. Adenocarcinoma was diagnosed preoperatively in all patients and classified as clinical stage IA (T1, N0) based on the findings of upper gastrointestinal endoscopy, upper gastrointestinal series, and abdominal contrast computed tomography (CT) according to the 3rd English edition of the Japanese classification of gastric carcinoma [11]. Eighty patients were judged to be unsuitable candidates for endoscopic submucosal resection (ESD) and 20 patients were judged to have undergone non-curative resection after ESD. During the study period, open distal gastrectomy with D1 or D1+ lymph node dissection was selected for seven patients with clinical stage IA cancer based on a history of upper abdominal surgery (*n* = 3), combined surgery with other types of cancer (*n* = 2), and severe co-morbidities (*n* = 2). This study was reviewed and approved by the institutional review board of Okayama University (No. 1505-024).

### Surgical procedure

**Fig. 1:**
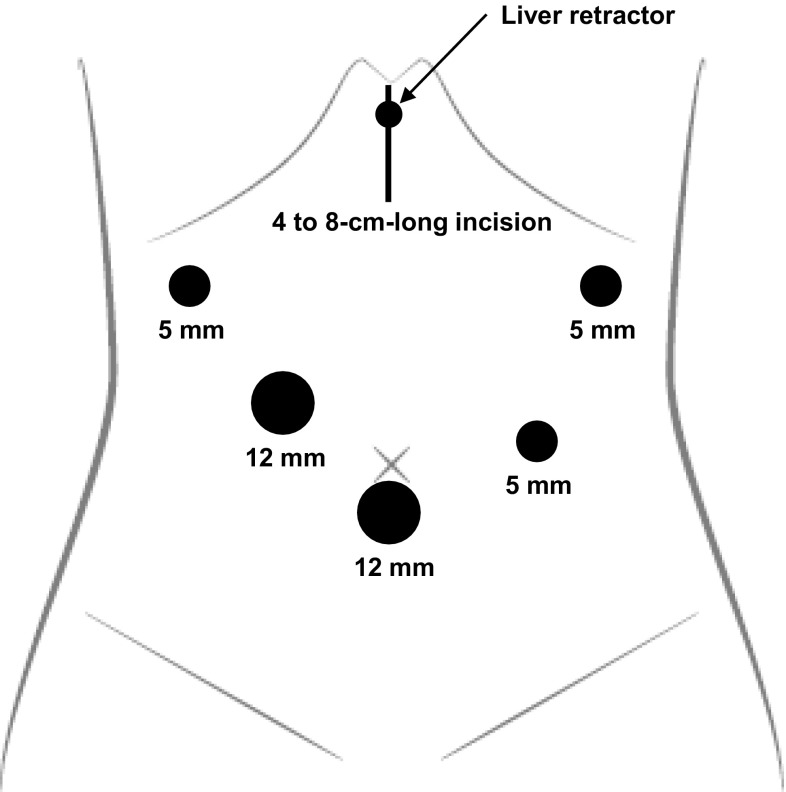
Port placement

LADG with curative lymph node dissection was performed according to the standardized procedure in all patients. Briefly, the first 12-mm trocar was inserted below the umbilicus using the open surgical method, and then four ports were inserted under laparoscopic guidance (Fig. 1). Pneumoperitoneum pressure was kept at around 10 mmHg. Following intraperitoneal observation, distal gastrectomy was started by dividing the greater omentum, from more than 3 cm from the gastroepiploic arcade toward the lower pole of the spleen, using ultrasonic coagulating shears. The left gastroepiploic artery (LGEA) and vein (LGEV) were exposed and divided after the omental branch bifurcated. The operator then moved to the left side of the patient. After the greater omentum was continuously divided toward the right side and the transverse mesocolon was adequately taken down, the right gastroepiploic vein (RGEV) was exposed and divided at the junction with the superior pancreaticoduodenal vein (ASPDV). The right gastroepiploic artery (RGEA) was then double-clipped and divided at the root from the gastroduodenal artery (GDA). The operator moved back to the right side of the patient, and the duodenum was transected using an endoscopic linear stapling device. The right gastric artery (RGA) and vein (RGV) were then exposed and divided at the root. For D1+ lymph node dissection, lymph nodes along the common hepatic artery (CHA) (station 8a) and the celiac artery (CA) (station 9) were dissected along the outermost layer of the nerve. After dividing the left gastric vein (LGV), the left gastric artery (LGA) was exposed, double-clipped, and divided at the root. The laparoscopic procedure was finished after the perigastric lymph node dissection along the lesser curvature. A midline skin incision of around 5 cm was made in the upper abdomen. After the distal two-thirds of the stomach were resected, Billroth-I reconstruction was performed using a circular stapler, or Billroth-II, or Roux-en-Y reconstruction was performed using a linear stapler. The minilaparotomy and trocar sites were closed after the placement of a closed suction drain around the anastomosis.

### Trainers and trainees

Seven operators were involved in this study, six of whom were trainees, and (after one of these six became qualified by the ESSQS as a trainer), two of whom were ESSQS-accredited trainers. Surgical experience as an operator varied greatly among the trainees. The median operator experience with open gastrectomy and laparoscopic cholecystectomy prior to starting LADG training was 25 cases (range 5–100 cases) and 20 cases (range 3–45 cases), respectively, and none of the trainees had operator experience with laparoscopic colectomy. The trainees were required to commit to improving their laparoscopic skills by practicing suturing in the box trainer and watching exemplary videos and their own surgical videos regularly. They were also encouraged to participate in training sessions, such as hands-on training using a porcine model, animal laboratory training, and an educational seminar organized by the Department of Gastroenterological Surgery of Okayama University Graduate School of Medicine and the Minimally Invasive Therapy Center of Okayama University Hospital. Trainers decided if the trainees were capable of performing LADG as an operator based on the trainees’ laparoscopic surgical skills and knowledge, regardless of operator experience with surgery, such as open gastrectomy, laparoscopic colectomy, and laparoscopic cholecystectomy, or experience as a first assistant or scopist for LADG.

### Clinical data

The patient characteristics we analyzed included age, sex, body mass index (BMI), presence of preoperative co-morbidity, and the American Society of Anesthesiologists physical status (ASA-PS) classification [12]. Histological findings were described according to the 3rd English edition of the Japanese Classification of Gastric Carcinoma [11]. Surgical outcomes included operation time, reconstruction method, skin incision length, extent of lymph node dissection, number of retrieved lymph nodes, presence or absence of concurrent cholecystectomy, blood loss, postoperative complications classified as grade I or higher on the Clavien–Dindo classification [13, 14], the number of days until the first flatus after surgery, the highest body temperature (BT) after surgery, duration of BT ≥37.5 °C, and the length of hospital stay after surgery.

### Learning curve of the trainees

We used the Mann–Whitney *U* test for continuous data and Pearson’s Chi squared test for categorical data to compare the characteristics of patients operated on by the trainees with those of the patients operated on by the qualified surgeons. Then, patients operated on by the trainees were divided into four groups depending on each operator’s experience (1–5, 6–10, 11–20, and more than 20 cases), and the average operation time was compared among the groups and then with that of those performed by the qualified surgeons. For the operations performed by the trainees whose operator experience was 20 cases or less, statistical analysis (Student’s *t* test) focused on the position of the trainer (first assistant or scopist) to investigate the importance of the trainer’s position for inexperienced trainees.

### Statistical analysis of good-quality operations

Assuming that surgical cases with an operation time <240 min, blood loss <50 ml, and retrieved lymph nodes ≥15 would be “good-quality operations”, we evaluated the independent factors for achieving “good-quality operations”. Pearson’s Chi square test was used to assess the influence of the following factors on achieving “good-quality operations”: age, sex, BMI, ASA-PS, presence of preoperative co-morbidity, number of operations performed by the operator, the extent of lymph node dissection, and the presence of cholecystectomy concurrently performed with LADG. Multivariate logistic regression analysis was done to assess the independent effects of important factors identified on the univariate analysis. A *p* value <0.05 was considered significant.

## Results

### Patients’ characteristics and histological findings (Table 1)

The median age of the patients was 66 years (range 29–85 years), and the male-to-female ratio was 6:4. The median BMI was 22.8 kg/m^2^ (range 16.9–32.5 kg/m^2^). A total of 53 patients (53%) had some co-morbidity at the time of surgery, such as hypertension, hyperlipidemia, diabetes mellitus, cardiovascular disease, respiratory disease, liver disease, renal disease, brain disease, or steroid use. The condition of nine patients (9%) was classified as class 3 or higher according to the ASA-PS classification.

**Table 1:** Patients’ characteristics and histological findings

Age (years)		
Median (range)	66	(29–85)
Sex		
Male	60	(60%)
Female	40	(40%)
Body mass index (BMI), kg/m^2^		
Median (range)	22.8	(16.9–32.5)
Preoperative co-morbidities		
Yes	53	(53%)
ASA-PS		
1	35	(35%)
2	56	(56%)
3 or more	9	(9%)
Histological type		
Differentiated	60	(60%)
Undifferentiated	40	(40%)
Histological T status		
T1	97	(97%)
T2	0	(0%)
T3	2	(2%)
T4	1	(1%)
Histological N status		
N0	90	(90%)
N1	6	(6%)
N2	3	(3%)
N3	1	(1%)
Histological stage		
IA	88	(88%)
IB	5	(5%)
IIA	4	(6%)
IIB	3	(2%)

While all patients had clinical T1 and N0 diagnosed before surgery, three (3%) had T3 or T4 diagnosed histologically after surgery, and ten (10%) had lymph node metastases. Four patients (4%) had disease classified into histological stage IIA or IIB, and three of these four patients received adjuvant chemotherapy with S-1 after surgery, according to the Japanese gastric cancer treatment guidelines 2010 (ver. 3).

### Short-term surgical outcomes (Table 2)

Seven operators, including two surgeons qualified by the ESSQS, performed LADG with curative D1/D1+ lymph node dissection for 100 patients with gastric cancer. The majority (*n* = 93) of patients underwent D1+ lymph node dissection, and Billroth-I was most frequently selected for the reconstruction procedure (*n* = 86; 86%). The median skin incision was 5 cm (range 4–10 cm) in length and Roux-en-Y tended to need a longer incision than Billroth-I (6 cm, range 4–8 vs. 5 cm, range 4–10 cm, respectively; *p* = 0.0767). Cholecystectomy was performed concurrently in 8 patients (8%). There were no conversions from LADG to open distal gastrectomy (ODG). The median operation time was 258.5 min (range 144–447 min). The median blood loss was 40 ml (range 0–790 ml) and blood transfusion was not required for any patients.

**Table 2:** Surgical outcomes

Operation time, min		
Median (range)	258.5	(144–447)
Reconstruction method		
Billroth-I	86	(86%)
Billroth-II	1	(1%)
Roux-en-Y	13	(13%)
Length of skin incision, cm		
Median (range)	5	(4–10)
Lymph node dissection		
D1	7	(7%)
D1+	93	(93%)
Retrieved lymph nodes		
Median (range)	28.5	(6–72)
Concurrent cholecystectomy		
Yes	8	(8%)
Blood loss		
Median (range)	40	(0–790)
Postoperative complications (Clavien–Dindo grade 1 or higher)		
Anastomotic leakage	1	(1%)
Pancreatic fistula	1	(1%)
Delayed gastric empting	2	(2%)
Wound infection	2	(2%)
Pneumonia	3	(3%)
Urinary tract infection	1	(1%)
Total	10	(10%)
First flatus, postoperative days		
Median (range)	2	(1–5)
Highest body temperature (BT), ^°^C		
Median (range)	38.0	(37.2–39.3)
Duration of BT ≥37.5 °C		
Median (range)	2	(0–13)
Hospital stay, days		
Median (range)	11	(9–81)

With regard to postoperative complications, grade III anastomotic leakage developed in one patient (1%), and a grade III pancreatic fistula developed in one patient (1%). Two patients (2%) suffered from delayed gastric emptying (grades I and II), which prolonged the hospital stay. Overall, postoperative adverse events (grade I or higher) were observed in 10 patients (10%), including two grade III events described above as the most severe complications. All patients passed flatus postoperatively after a median period of 2 days (range 1–5 days). The median highest BT was 38.0 °C (range 37.2–39.3 °C), and the median duration of BT ≥37.5 °C was 2 days (range 0–13 days). The median length of hospital stay after surgery was 11 days (range 9–81 days).

### Learning curve of the trainees and factors for good quality operations

**Fig. 2:**
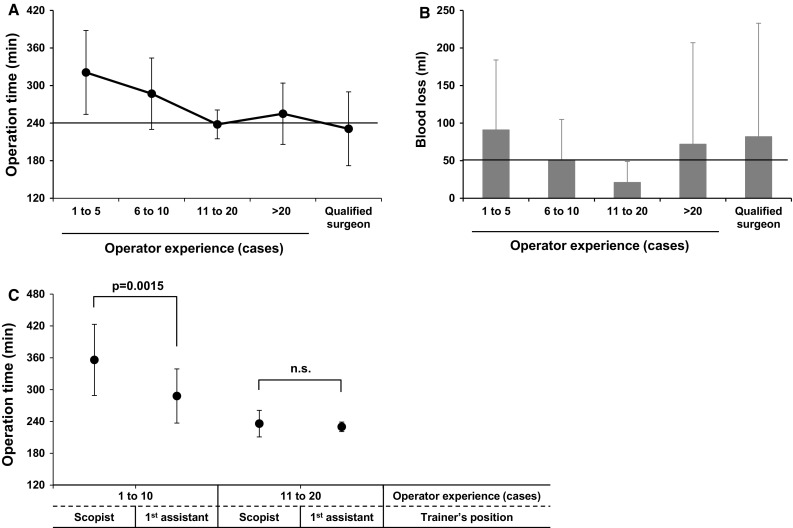
Learning curve of the trainees. **a** Average operation time for laparoscopy-assisted distal gastrectomy (LADG) performed by the trainees, divided into four groups depending on each operator’s experience (1–5, 6–10, 11–20, and more than 20 cases), was compared among the groups and also with that of operations performed by qualified surgeons. **b** Same comparison was done for average blood loss. **c** For the operations done by trainees with operator experience of 20 cases or less, the influence of the trainer’s position, as the first assistant or scopist, on operation time was evaluated

To assess the learning curve of the trainees, operations performed by the trainees were divided into four groups according to operator experience (1–5, 6–10, 11–20, and more than 20 cases), which were compared with each other and with operations performed by the qualified surgeons, in terms of the average operation time (Fig. 2a) and the average blood loss (Fig. 2b). The qualified surgeons tended to perform operations for patients with preoperative co-morbidities (*p* = 0.0569) and higher ASA-PS (*p* = 0.1922; Table 3). The average operation time for the trainees plateaued at around 240 min after 10 cases, which was comparable to that of the qualified surgeons. The average operative blood loss was similar for the trainees and the qualified surgeons. Furthermore, when the average operation time for the trainees was evaluated focusing on the difference in the trainer’s position (the first assistant or scopist), for operations performed by the trainees whose experience was 1–10 cases, the average operation time was significantly longer when the trainer was the scopist vs when the trainer was the first assistant. For operations performed by the trainees whose experience was 11–20 cases, there was no difference in operation time depending on the trainer’s position (Fig. 2c).

**Table 3:** Characteristics of patients operated on by the trainees vs. those operated on by the qualified surgeons

	Trainees (*n* = 79)	Qualified surgeons (*n* = 21)	*p* value
Age, years			
Median (range)	65 (35–85)	71 (29–84)	0.2956
Sex			
Male	45 (57%)	15 (71%)	0.2291
Female	34 (43%)	6 (29%)	
Body mass index (BMI), kg/m^2^			
Median (range)	22.8 (17.0–32.5)	22.7 (16.9–31.9)	0.3432
Preoperative co-morbidities			
Yes	38 (48%)	15 (71%)	0.0569
ASA-PS			
1	31 (39%)	4 (19%)	0.1922
2	42 (53%)	14 (67%)	
3 or more	6 (8%)	3 (14%)	

Finally, we assumed that operations that took <240 min with blood loss <50 ml and retrieved lymph nodes ≥15 were “good-quality operations”. Thus, we analyzed the important factors for achieving “good-quality operations” (Table 4). Univariate and multivariate analyses showed that >10 operator cases was the independent and most critical factor for achieving “good-quality operations” (*p* < 0.0001, odds ratio 13.3), followed by BMI (<22 kg/m^2^) (*p* = 0.0117, odds ratio 3.46).

**Table 4:** Univariate and multivariate analyses of factors related to a good-quality operation (operation time <240 min, blood loss <50 ml, retrieved lymph nodes ≥15)

	Univariate	Multivariate	
	*p* value	Odds ratio	*p* value
Age			
<60/≥60 years	0.5481		
Sex			
Male/female	0.4962		
BMI			
<22/≥22 kg/m^2^	0.0022	3.46	0.0117
<25/≥25 kg/m^2^	0.0068		
ASA-PS			
1/2, 3	0.8613		
Preoperative co-morbidities			
No/yes	0.7010		
Operator experience (cases)			
>10/≤10	0.0003	13.3	<0.0001
>20/≤20	0.0446		
Lymph node dissection			
D1/D1+	0.6711		
Reconstruction method			
B-I/B-II, RY	0.0680	3.33	0.1316
With cholecystectomy			
Yes/no	0.1488	0.12	0.0309

## Discussion

In a multicenter, prospective, phase II clinical trial in Japan (JCOG 0703), the incidence of anastomotic leakage or pancreatic fistula, which was the primary endpoint in this study, was only 1.7% (3/176). This incidence was lower than the estimate expected from the previous retrospective studies, in which anastomotic leakage and pancreatic fistula were observed in 1.7–14% and 1.0%, respectively, and it was equivalent to the incidence after ODG, in which anastomotic leakage and pancreatic fistula reportedly occurred in 0.6–2.7% and 0.6%, respectively [4]. In another multicenter, prospective, clinical trial conducted in Korea (KLASS trial), the incidence of anastomotic leakage was 1.7% (3/172) [15]. One of the possible reasons for the good outcomes of these prospective trials in Japan and Korea may be that the attending surgeons for these clinical trials, all had much experience with LADG and ODG, of “more than 30 LADGs and 30 ODGs” in the JCOG 0703 and “more than 50 LADGs and 50 ODGs” in the KLASS trial. The reason why such a strict limitation on operator experience was set in these clinical trials was because LADG requires more advanced surgical and management skills than ODG and critical complications can be caused by this operation being performed by inexperienced surgeons.

In Japan, the ESSQS was established in 2001 by the JSES with the aim of improving endoscopic techniques and reducing complications related to endoscopic procedures. To be eligible for accreditation, applicants are strictly assessed on their endoscopic surgical technique by unedited video, in addition to a series of documents. Although the overall success rate of applicants assessed through this system is relatively low, at less than 50%, the incidence of complications has been shown to be significantly lower in patients treated by accredited surgeons than by failed surgeons, which means that the ESSQS has a positive influence on improving and standardizing endoscopic surgery [16]. Since the introduction of laparoscopic surgery to an institute is undoubtedly challenging and demanding, a meaningful training system was reported by Kinoshita et al. That was the “LADG Basic Lab Course”, a 1-day professional training course held for a team of surgeons and operating nurses from 20 different centers in Japan, designed to help the participants with the smooth introduction of LADG to each institution [17]. In two prospective feasibility studies of LADG, a solid background in open gastrectomy and laparoscopic surgery, along with an adequate training system, was reported to be a key to the successful and safe implementation of LADG [6, 7]. Mochizuki et al. required surgical experience with more than 50 conventional gastrectomies and 30 laparoscopic cholecystectomies, and Yoshikawa et al. more strictly required more than 300 open gastrectomies, more than 100 laparoscopic cholecystectomies, more than 5 laparoscopic colectomies, and more than 5 laparoscopic partial gastrectomiesin their study. With regard to the learning curves of LADG, although many reports discussed focusing on only the number of cases of operator experience, Nunobe et al. noted the importance of training as the first assistant and scopist before the first case as an operator [9, 18–20]. Based on a large database of 788 LADG cases, they reported that trainees reached the trainer’s level within only six cases in terms of the average operation time, if trainees performed their first LADG as an operator after sufficient experience with 45 cases as the first assistant and 41.4 cases as scopist.

In the present retrospective study of 100 consecutive LADG cases, there was one case of anastomotic leakage (1%) and one case of pancreatic fistula (1%). Although the outcomes of a retrospective study should not be compared with those of large-scale prospective studies, the present short-term LADG outcomes would be considered acceptable, given the fact that inexperienced trainees performed LADG in many patients, unlike in the multicenter, prospective studies performed in Japan and Korea, and the fact that there were more high-risk patients included in this study. Furthermore, it is safe to assume that this was accomplished by our established training system, as evidenced by the result that the learning curve of the trainees, who acquired the entire standardized procedures of LADG, plateaued after 10 cases as an operator, despite less operator experience with open gastrectomy and laparoscopic cholecystectomy before starting the LADG training, with quality comparable to that of qualified surgeons in terms of operation time and blood loss.

This retrospective study revealed an additional finding that may improve our training system. It is a difficult but important decision for a trainer to take the role of scopist assuming that the first assistant is sufficiently proficient to support an inexperienced operator. This study included 16 operations performed by inexperienced surgeons whose operator experience was 20 cases or less, in which the trainer took the role of scopist. In these 16 cases, the average operation time was significantly longer when surgery was performed by the trainees whose operator experience was 10 or less, although the experience level of the first assistant, defined by operator experience with LADG, did not influence the operation time. This finding suggested that the trainer should be the first assistant when the trainee’s experience is 10 cases or less.

The safety of LADG is currently being confirmed by JCOG 0703, a phase III, multicenter clinical trial (JCOG 0912) designed to demonstrate the non-inferiority of LADG to ODG in terms of overall survival. If the non-inferiority of LADG to ODG is confirmed by this study, LADG will be described in the guideline as a standard procedure for the early gastric cancer. However, in these clinical trials, the LADG procedures were performed only by experienced surgeons; therefore, we need to establish a system that enables us to provide good-quality operations, equivalent to these clinical trials, even when inexperienced surgeons perform LADG as operators. To accomplish this, standardization of the LADG procedure and establishment of a training system in each hospital are inevitable. From this perspective, our LADG procedure and training system are appropriate, although further efforts toward a better quality operation based on the results of this study are obviously necessary.
